# Resilience, Hope, and Subjective Happiness Among the Turkish Population: Fear of COVID-19 as a Mediator

**DOI:** 10.1007/s11469-020-00443-5

**Published:** 2020-12-03

**Authors:** Seydi Ahmet Satici, Ahmet Rifat Kayis, Begum Satici, Mark D. Griffiths, Gurhan Can

**Affiliations:** 1 Department of Psychological Counselling, Artvin Coruh University, Artvin, Turkey; 2 Department of Psychological Counselling, Kastamonu University, Kastamonu, Turkey; 3 International Gaming Research Unit, Psychology Department, Nottingham Trent University, 50 Shakespeare Street, Nottingham, NG1 4FQ UK; 4 Department of Psychological Counselling, Hasan Kalyoncu University, Gaziantep, Turkey

**Keywords:** COVID-19, Subjective happiness, Resilience, Hope, Fear of COVID-19

## Abstract

Psychology deals with not only mental disorders but also psychological strengths within individuals. Psychological strengths will play an important role in struggling with the global novel coronavirus 2019 (COVID-19) pandemic. The present study tested a model concerning the relationship between resilience, hope, and subjective happiness using structural equation modeling to identify the mediating role of fear of COVID-19. A cross-sectional survey was conducted among a convenience sample of 971 Turkish individuals (aged 18 to 74 years) from 75 of 81 cities in Turkey. The survey included the Subjective Happiness Scale, Fear of COVID-19 Scale, Brief Resilience Scale, and the Dispositional Hope Scale, and data were analyzed using structural equation modeling (SEM). The SEM demonstrated an association between resilience–hope and subjective happiness was mediated by fear of COVID-19 (CMIN/*df* = 2.664, CFI = 0.994, NFI = 0.984, TLI = 0.984, GFI = 0.994, RMSEA = 0.044, SRMR = 0.024, AIC = 81.334, ECVI = 0.084). Resilience had a direct effect and an indirect effect on subjective happiness via fear of COVID-19. Hope also had a direct effect and an indirect effect on subjective happiness via fear of COVID-19. Consequently, in the fight against COVID-19, individuals who are resistant to stress and have a belief that they can find a way to cope can help prevent the fear of COVID-19 and so enhance good mental health.

## Introduction

The novel coronavirus disease 2019 (COVID-19) pandemic is a serious health threat that (at the time of writing) has infected more than 43 million people worldwide and resulted in the death of more than 1.1 million individuals (World Health Organization 2020). From March 11, when the first case of COVID-19 was reported in Turkey, to October 30, over 370,800 cases had been reported and over 10,000 individuals had died from it (Republic of Turkey Ministry of Health 2020). Turkey’s government enforced many measures to mitigate the spread of COVID-19, such as enforcing spatial distancing, the use of medical face masks, closing schools, encouraging self-isolation, and quarantining individuals returning from abroad. With COVID-19 having spread globally, the absence of a scientifically proven cure, and the difficulty in controlling it, the resulting situation has led to devastating economic, social, and psychological effects.

### COVID-19 and Psychological Impact

One of the most important issues regarding COVID-19 is preventing adverse psychological effects among individuals. Research carried out at the start of the pandemic in China showed that symptoms of depression, anxiety, stress, and panic disorders had increased compared to levels prior to the COVID-19 outbreak (Qiu et al. 2020; Wang et al. 2020). COVID-19 can also trigger panic and fear among some individuals (Ahorsu et al. 2020). Such feelings can arise from the fear of being infected, infecting others, dying, and/or losing loved ones. Additionally, concerns such as not being able to receive the necessary medical care or the possibility of losing one’s job as a result can also weigh heavily on the mind of the individual (Montemurro 2020; Ornell et al. 2020; Pakpour and Griffiths 2020).

Another possible reason that may cause fear and panic relating to COVID-19 is the measures taken to inhibit the spread of the virus such as spatial distancing, self-quarantine, and restricting time outdoors (Anderson et al. 2020; Wilder-Smith et al. 2020). These measures have forced individuals to stay at home leading to a restriction of their social relationships. Consequently, this has led to individuals being deprived of social support, which is a likely protective factor in terms of fear of COVID-19 as a means of coping (Chou 2000; Kassam 2019). The continual following of news and developments concerning COVID-19 (especially via social media which may include misinformation) is also a factor that risks increasing fear of COVID-19 (Ahorsu et al. 2020). Additionally, although fear has strengths such as sensitizing and persuading dangerous situations, it can also cause maladaptive coping behaviors if the level of fear is too great (Witte and Allen 2000). Therefore, the importance of psychological protective factors cannot be understated in regard to the fear of COVID-19.

### Resilience as a Factor in Preventing Poor Mental Health

One of the demonstrable protective factors in preventing psychological disorders is resilience. Resilience is the speedy recovery and the ability to return to daily functioning, after having experienced stressful life events that have resulted in functional breakdown (Carver 1998). In other words, resilience can be viewed as the ability to stay strong during challenging times (Jackson et al. 2007). During COVID-19 pandemic, the resilience of individuals may have decreased while their vulnerability may have increased (Sadati et al. 2020). This is why resilience can be considered as an important component in coping with the fear, panic, anxiety, and stress resulting from COVID-19. The finding that resilience interventions help in the creation of effective coping strategies for stress supports this view (Steinhardt and Dolbier 2008). Alongside this, resilience has positive associations with variables such as good psychological well-being (Sagone and De Caroli 2014; Souri and Hasanirad 2011), subjective well-being (Bajaj and Pande 2016), and subjective happiness (Choi and Kim 2018), all of which are indicators of good mental health. Furthermore, it has also been posited that resilience is not only protective in terms of mental health but also economically and biologically (Djalante et al. 2020). Consequently, strong resilience is likely to be associated with a low fear of COVID-19.

### Hope as a Factor in Preventing Poor Mental Health

Hope is the belief that the paths to reach one’s goals can be found and the motivation in trying out those different paths (Snyder et al. 2002). From this perspective, a hopeful individual, even under challenging life conditions, has the strength to find alternative solutions and to apply them. Consequently, hope can be seen as a protective factor in mental well-being in relation to the difficult conditions created by COVID-19. The COVID-19 pandemic has forced individuals to adopt many new modes of behavior in relation to areas such as personal hygiene, working conditions, and social relationships (Ahorsu et al. 2020; Anderson et al. 2020). In light of this, if it is perceived that having to develop new modes of behavior as protection against COVID-19 as a goal, then it is posited that hope will be a strong factor when trying to attain such a goal. One aspect of hope is that it encompasses positive expectations for the future (Snyder 2000), so naturally an individual may have hopeful expectations that COVID-19 will pass. This motivates the individual into making effort regarding both the present and the future. Moreover, there are findings demonstrating that hope has a negative association with psychological disorders (Glass et al. 2009; Rawdin et al. 2013). Similarly, interventions programs that facilitate hope decrease psychological symptoms (Rustøen et al. 2011). On the other hand, hope has a positive association with subjective happiness (Sariçam 2015) and subjective well-being (Kato and Snyder 2005; Satici 2016; Shenaar-Golan 2017; Yalçın and Malkoç 2015). When these findings are taken as a whole, hope can be thought of as playing a protective role that stimulates good mental health and counteracts psychological problems caused by COVID-19.

### Happiness and Good Mental Health

One of the indicators of good mental health is subjective happiness, and the fear of COVID-19 (discussed in the next section) has the potential to affect an individual’s subjective happiness. Subjective happiness is the balance of positive and negative feelings and satisfaction in one’s life (Diener et al. 2009). In essence, the more that an individual experiences more positive than negative feelings, and the more satisfaction they have in their life, the greater they will feel happiness. However, infectious diseases have a negative effect on happiness (Lau et al. 2008). Therefore, it can be posited that the fear of COVID-19 will decrease happiness among individuals and impact their mental health negatively. Many studies have in fact pointed to the possibility of COVID-19 causing psychological disorders (e.g., Holmes et al. 2020; Mucci et al. 2020). Alongside this, positive psychology-based interventions programs have been found to decrease fear and increase happiness (Lambert et al. 2019). This finding supports the notion that fear of COVID-19 will have a negative correlation with subjective happiness. Also, factors thought to help prevent fear of COVID-19, such as resilience (Choi and Kim 2018; Yildirim and Belen 2019) and hope (Aghababaei et al. 2016; Satici 2016), have a positive correlation with subjective happiness.

### Fear of COVID-19

Fear is a natural reaction in the face of danger. For this reason, it is inevitable that fear is experienced in relation to COVID-19. In fact, fear can be a source of motivation that leads individuals to take precautions against dangerous situations such as the COVID-19 pandemic (Nabi and Myrick 2019). However, the uncertainty surrounding the dangers of COVID-19 means that fear can become both chronic and disproportionate (Mertens et al. 2020). The Fear of COVID-19 Scale (FCVS-19) developed by Ahorsu et al. (2020) has quickly become a widely used instrument to assess the fear of COVID-19. The FCVS-19 has already been adapted to many different languages including Arabic (Alyami et al. 2020), Bangla (Sakib et al. 2020), English (Harper et al. 2020 [in the UK]; Winter et al. 2020 [in New Zealand]); Hebrew (Bitan et al. 2020), Italian (Soraci et al. 2020), Japanese (Masuyama et al. 2020), Malay (Pang et al. 2020), Portuguese [in Brazil] (Abad et al. 2020), Russian (Reznik et al. 2020), and Turkish (Satici et al. 2020) which has stimulated much of research on fear of COVID-19. These many studies in such a short period of time show that the fear of COVID-19 is being researched worldwide in terms of examining the psychological effects of fear in the COVID-19 pandemic.

### The Present Study

To date, the world has not experienced a widespread virus that has impacted on the world the way that COVID-19 has. The first COVID-19 case in Turkey was reported on March 11, 2020, and since then, many measures have been taken to isolate and try and control the speed at which the virus is spreading. These measures have had a negative impact on economy and social lives as well as creating fear, panic, and anxiety. That is why it is crucial to determine the factors that protect the mental health of individuals and their daily functioning.

Focusing on the strengths of a person instead of psychological disorders is essential in terms of protective and preventive mental health services. It therefore follows that in the battle against the negative outcomes of COVID-19, focusing on the individual’s strengths is an important factor in preventing the emergence of psychological disorders. In this context, both hope (Griggs 2017; Trezise et al. 2018) and resilience (Reyes et al. 2019; Shin et al. 2019) as protective factors in helping prevent psychological disorders developing will play important roles in countering the negative effects of COVID-19.

Studies that have evaluated the effects COVID-19 from a mental health perspective, commonly draw attention to psychological disorders that it may lead to (e.g., Conversano et al. 2020; Qiu et al. 2020; Zhang et al. 2020). However, it is argued that focusing on indicators that comprise a healthy psychological outlook such as resilience, hope, well-being, and happiness is more effective in developing good mental health (Peterson 2006; Peterson and Seligman 2004). In this respect, examining the association between fear of COVID-19 and subjective happiness (which is a psychological strength) can provide important information on how individuals can become stronger mentally during the COVID-19 pandemic.

**Fig. 1:**
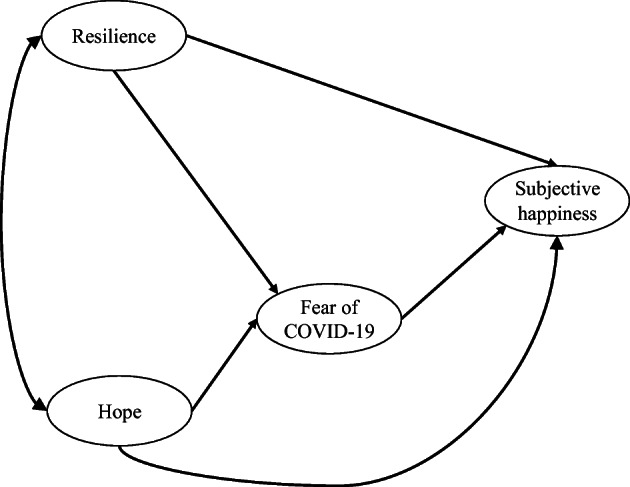
The model of the proposed relationships between the study variables

Consequentially, the protective factors associated with overcoming the fear of COVID-19 should be examined, not just the psychological disorders that it may lead to. Alongside this, another area necessitating examination concerns what the protective factors and what might happen if an individual’s strengths are weakened. In relation to the fear of COVID-19, previous research has not examined resilience and hope as protective factors, or subjective happiness as an indicator of good mental health. Therefore, the role of fear of COVID-19 as a mediator between resilience, hope, and subjective happiness was tested in the present study. Consequently, two hypotheses (Hs) were formulated based on the aforementioned literature: (H_1_) the relationship between resilience and subjective happiness will be mediated by fear of COVID-19 and (H_2_) the relationship between hope and subjective happiness will be mediated by fear of COVID-19. A graphical model of the hypothesized relationships is shown in Fig. 1:

## Method

### Participants and Procedure

The study sample comprised 971 Turkish volunteers consisting of 711 females (73.2%) and 260 males (26.8%). Their ages ranged from 18 to 74 years (*M* = 24.46 years, SD = 7.38). Data were obtained from a cross-sectional study using convenience sampling in the first week of May 2020. A web-based questionnaire was created and distributed using a link on Turkish social networking sites. After giving brief information about the study, informed consent was obtained from the participants. Participants completed four self-report measures which were anonymous to avoid biased responses. The web-based questionnaire was designed so that participants could withdraw at any time. Additionally, when they wanted to submit the survey, they were asked if they could complete all the items. Therefore, there were no missing data among the surveys that were submitted.

### Measures

#### Subjective Happiness

This was assessed using the Subjective Happiness Scale (SHS; Lyubomirsky and Lepper 1999), a four-item self-report scale with items (e.g., To what extent does this characterization describe you? “Some people are generally very happy. They enjoy life regardless of what is going on, getting the most out of everything”) responded to on a scale from 1 (*not at all*) to 7 (*a great deal*). The total score ranges between 4 and 28, with lower scores indicating poorer subjective happiness. Adaptation of the SHS into Turkish was carried out by Akin and Satici (2011) and has very good internal consistency reliability (*α* = .86), as well as good structure validity (Akin and Satici 2011). In the present study, the reliability coefficients were good (*α* = .74 and *ω* = .75).

#### Fear of Coronavirus-19

This was assessed using the Fear of COVID-19 Scale (FCVS-19; Ahorsu et al. 2020), a seven-item self-report scale with items (e.g., “I cannot sleep because I’m worrying about getting coronavirus-19”) responded to on a scale from 1 (*strongly disagree*) to 5 (*strongly agree*). The total score ranges between 7 and 35, with higher scores indicating greater fear of COVID-19. Adaptation of the FCVS-19 into Turkish was carried out by Satici et al. (2020) and has very good internal consistency reliability (*α* = .85), as well as good structure validity (Satici et al. 2020). In the present study, the reliability coefficients were very good (*α* = .85 and *ω* = .85).

#### Resilience

This was assessed using the Brief Resilience Scale (BRS; Smith et al. 2008), a six-item self-report scale with items (e.g., “I tend to bounce back quickly after hard times”) responded to on a scale from 1 (*strongly disagree*) to 5 (*strongly agree*). The total score ranges between 6 and 30, with lower scores indicating poorer resilience. Adaptation of the BRS into Turkish was carried out by Doğan (2015) and has very good internal consistency reliability (*α* = .83), as well as good structure validity (Doğan 2015). In the present study, the reliability coefficients were very good (*α* = .81 and *ω* = .81).

#### Hope

This was assessed using the Dispositional Hope Scale (DHS; Snyder et al. 1991), a 12-item self-report scale with items (e.g., “I can think of many ways to get out of a jam”) responded to on a scale from 1 (*definitely false*) to 8 (*definitely true*). The DHS comprises two subscales: agency and pathway. Adaptation of the BRS into Turkish was carried out by Tarhan and Bacanlı (2015) and has very good internal consistency reliability (*α* = .86), as well as good structure validity (Tarhan and Bacanlı 2015). In the present study, the reliability coefficients were very good (*α* = .87 and *ω* = .87).

### Data Analysis

First, bivariate correlations (Pearson’s coefficient) were calculated. Second, descriptive statistics, convergent and discriminant validity, and internal reliabilities were examined. Third, a mediation model analysis based on a two-step approach SEM (measurement and structural model) recommended by Anderson and Gerbing (1988) was performed. Maximum likelihood estimation was used in the SEM. The following model fit indices were examined: root mean square error of approximation (RMSEA), standardized root mean square residual (SRMR), comparative fit index (CFI), normed fit index (NFI), Tucker–Lewis index (TLI), and goodness of fit (GFI) such that RMSEA and SRMR values ≤ .08, CFI, NFI, TLI, and GFI values ≥ .90 generally represent acceptable fit to the observed data (Hu and Bentler 1999; MacCallum et al. 1996). In addition, the parceling technique that is used to reduce measurement errors in single factor measurement (Little et al. 2002) was also included in the SEM. Therefore, the uni-dimensional SHS, FCVS-19, and BRS were divided into two parcels. Data were analyzed utilizing IBM SPSS Statistics 22, AMOS 24, and JASP 0.11.1.0.

### Ethics

The study procedures were carried out in accordance with the Declaration of Helsinki and were approved by the Artvin Coruh University Scientific Research and Ethical Review Board (REF = E.5374) and Ministry of Health (Document Name: 2020-05-07T22-10-45). All participants provided informed consent and were informed that they could withdraw at any time from the study.

## Results

### Relationships Between the Variables

Table 1 shows that subjective happiness was negatively correlated with fear of COVID-19 (*r* = − .25, *p* < .001). Fear of COVID-19 was negatively correlated with resilience (*r* = − .42, *p* < .001) and hope (*r* = − .24, *p* < .001). Subjective happiness was positively correlated with resilience (*r* = .41, *p* < .001) and hope (*r* = .34, *p* < .001).

**Table 1:** Correlations and discriminant validity

Variable	1	2	3	4
1. Subjective happiness	*.76*			
2. Fear of COVID-19	− .25	*.90*		
3. Resilience	.41	− .42	*.74*	
4. Hope	.34	− .24	.48	*.84*

Diagonals (in italic) represent square root of AVE while off diagonals represent correlations

### Structural Equation Modeling

#### Measurement Model

The measurement model included four latent variables (subjective happiness, fear of COVID-19, resilience, and hope) and eight observed variables. All the fit indices for the measurement model indicated that they were a suitable fit to the data: RMSEA = 0.041, SRMR = 0.021, CMIN/*df* = 2.667, CFI = 0.993, NFI = 0.988, TLI = 0.985, GFI = 0.991. To test the measurement model, composite reliability (CR) and discriminant validity were also checked. Table 2 shows the factor loadings, mean, standard deviation, composite reliability (CR), average variance extract (AVE), and reliability coefficient (Cronbach’s *α* and Mc Donald’s *ω*) used to check the convergent validity of constructs. The results showed that factor loadings were between 0.72 and 0.94 and all of them were statistically significant. In addition, the results indicated that CRs were higher than 0.7 and the AVEs were greater than 0.5. The model had adequate convergent and discriminant validity according to the criteria proposed by Bagozzi and Yi (1988) and Fornell and Larcker (1981). All the reliability coefficients (*α* ≥ .74 and *ω* ≥ .75) were good. Consequently, these results demonstrated that the observed variables were strong representatives of the latent constructs.

**Table 2:** Factor loadings, descriptive statistics, CR, AVE, and reliabilities

Latent variables	Indicator	Loadings	*M*	SD	CR	AVE	*α*	*ω*
Subjective happiness	SHPar 1	.80	6.42	1.69	.75	.60	.738	.746
	SHPar 2	.76	8.10	1.74				
Fear of COVID-19	FCPar 1	.94	10.36	3.23	.90	.82	.845	.847
	FCPar 2	.87	7.98	2.58				
Resilience	RSPar 1	.72	9.85	2.38	.70	.54	.805	.807
	RSPar 2	.76	9.76	2.61				
Hope	Agency	.79	24.73	4.14	.82	.70	.865	.867
	Pathway	.88	23.22	4.31				

*SHPar* parcels of subjective happiness, *FCPar* parcels of fear of COVID-19, *RSPar* parcels of resilience, *CR* composite reliability, *AVE* average variance extract

#### Structural Model

To test the hypotheses, the partial mediation model was tested and compared to the full mediation model. The partial mediation model indicated an acceptable fit to the data (CMIN/*df* = 2.664, CFI = 0.994, NFI = 0.984, TLI = 0.984, GFI = 0.994, RMSEA = 0.044, SRMR = 0.024, AIC = 81.334, ECVI = 0.084). Compared to the partial mediation model, the fit of the full model yielded worse fit indices (CMIN/*df* = 7.875, CFI = 0.965, NFI = 0.960, TLI = 0.939, GFI = 0.969, RMSEA = 0.084, SRMR = 0.083, AIC = 165.999, ECVI = 0.171), and its CMIN/*df*, RMSEA, and SRMR values were above the reference values that indicate an acceptable fit. The partial mediation model was preferred because the AIC and ECVI coefficients were lower than the full mediation model’s AIC and ECVI coefficients. In addition, the chi-square test indicated that the partial mediation model provided a better fit to the data than the full mediation model (Δ*χ*^2^ = 88.67, *df* = 2, *p* < .001). In the partial mediation model, Cohen’s effect size was calculated for power analysis and a large effect size was determined (Cohen’s *f*^2^ = .421).

**Fig. 2:**
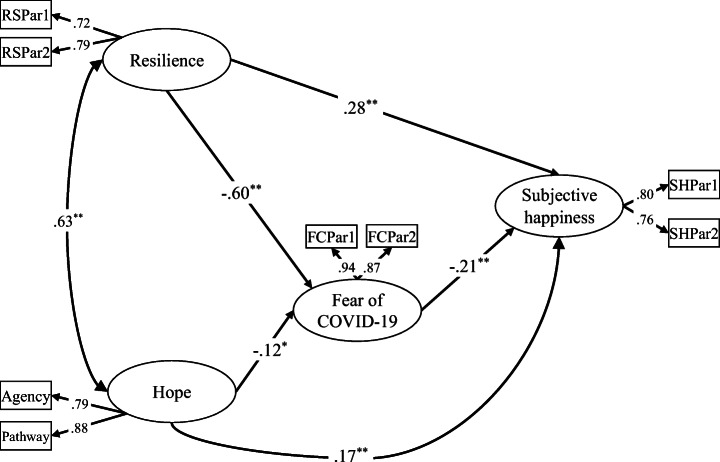
Standardized factor loadings for the structural model. *N* = 971; *p < .05, **p < .01

The partial mediation model showed that higher resilience predicted lower fear of COVID-19 (*γ* = − 0.60, *p* < .01) and higher subjective happiness (*γ* = 0.28, *p* < .01). In addition, a higher level of hope was associated with a lower fear of COVID-19 (*γ* = − 0.12, *p* < .05) and a higher level of subjective happiness (*γ* = 0.17, *p* < .01). As expected, a higher fear of COVID-19 predicted lower subjective happiness (*γ* = − 0.21, *p* < .01). Finally, the relationship between resilience and subjective happiness was partially mediated by fear of COVID-19. The bias corrected estimation of the standardized indirect effect was 0.126 (*p* < .01; BCa95% lower limit = − .701 to upper limit = − .521). Also, the relationship between hope and subjective happiness was partially mediated by fear of COVID-19 (0.024, *p* < .05; BCa95% lower limit = .037 to upper limit = .219). Taken together, this analysis demonstrated that resilience and hope had a direct positive influence on subjective happiness and indirectly via its negative effect on fear of COVID-19. All standardized factor loadings are presented in Fig. 2. Also, the indirect and total effects for bootstrapping are presented in Table 3.

**Table 3:** Indirect and total effects on subjective happiness

	Coefficient	95%CI	
		LL	UL
Resilience → fear of COVID-19 → subjective happiness	.126	.074	.187
Total effect (resilience on subjective happiness)	.410	.049	.513
Hope → fear of COVID-19 → subjective happiness	− .024	− .056	− .009
Total effect (hope on subjective happiness)	.146	.049	.240

Based on 5000 bias-corrected bootstrap sampling

*CI* confidence interval, *LL* lower limit, *UL* upper limit

## Discussion

Resilience and hope are protective factors in preventing mental disorders from developing (Griggs 2017; Reyes et al. 2019) and have a positive association with increased subjective happiness (Satici 2016). Consequently, resilience and hope are considered as protective factors helping to prevent damage to mental health that may arise from the fear of COVID-19. Therefore, this research tested the role of the fear of COVID-19 as a mediator in the relationship between resilience, hope, and subjective happiness.

The first finding demonstrated that fear of COVID-19 partially mediated the relationship between resilience and subjective happiness supporting H_1_. For this reason, in preventing individuals from developing a fear of COVID-19, resilience helps to protect their subjective happiness. During the COVID-19 pandemic, individuals have developed symptoms relating to fear, anxiety, depression, and stress (Ahorsu et al. 2020; Qiu et al. 2020; Wang et al. 2020). Eye movement desensitization and reprocessing group therapy carried out for the purpose of increasing resilience has been found to reduce fear (Zaghrout-Hodali et al. 2008). Resilience intervention has also been shown to help individuals developing effective coping strategies (Steinhardt and Dolbier 2008). Therefore, it can be posited that the development of resilience helps in coping with the fear and anxiety caused by COVID-19. Furthermore, previous studies have shown that resilience has a positive association with good psychological well-being (Sagone and De Caroli 2014; Souri and Hasanirad 2011) and subjective well-being (Bajaj and Pande 2016), which are widely used to define happiness alongside subjective happiness (Choi and Kim 2018). Consequently, the findings in the present study are consistent with the findings of previous research.

The second finding demonstrated that the relationship between hope and subjective happiness was partially mediated by fear of COVID-19 supporting H_2_. In other words, those individuals who had a high level of hope experienced low levels of fear of COVID-19, which resulted in protecting their subjective happiness. Just as in previous global pandemics (Mak et al. 2010; Wu et al. 2005), the experiences undergone during COVID-19 have caused post-traumatic stress disorder (PTSD) among a minority of individuals (Bo et al. 2020; Huang et al. 2020). However, hope has a negative association with PTSD and general psychological disorders (Glass et al. 2009; Rawdin et al. 2013). Moreover, interventions directed towards developing hope have been effective in decreasing the development of psychological disorders (Rustøen et al. 2011) and PTSD (Yousefi et al. 2016). On the other hand, hope has a positive correlation with subjective happiness (Sariçam 2015) and subjective well-being (Kato and Snyder 2005; Satici 2016; Shenaar-Golan 2017; Yalçın and Malkoç 2015). Thus, the finding here that hope appears to develop mental health by decreasing fear can be said to exhibit similar features with the findings of previous research.

Finally, resilience and hope, with a low level fear of COVID-19 as a mediator, were shown to predict a high level of subjective happiness. Therefore, with resilience and hope preventing fear of COVID-19 from reaching a level that disrupts daily functioning, the notion that they are helpful in protecting mental health appears feasible. The positive psychology approach posits that resilience and hope are psychological strengths and that instead of treating psychological disorders, developing psychological strengths is an alternative route in protecting mental health (Peterson 2006; Peterson and Seligman 2004). In fact, it has been demonstrated that intervention programs focused on positive psychology decrease fear and increase happiness (Lambert et al. 2019). Moreover, resilience (Choi and Kim 2018; Yildirim and Belen 2019) and hope (Aghababaei et al. 2016; Satici 2016) predict positive subjective happiness. Consequently, the findings here are in alignment with the theoretical knowledge concerning the aforementioned positive psychology, in addition to experimental and cross-sectional research findings (e.g., Lambert et al. 2019; Satici 2016).

### Implications

The findings of the present study fundamentally point to the development of strong psychological attributes being able to assist in the protection of mental health from the fear of COVID-19. Attributes such as resilience and hope can be increased with psychological intervention programs (Davidson et al. 2012; Ritchie et al. 2014; Rosenberg et al. 2015). It is within this context that the development and provision of resilience and hope building intervention programs are recommended as preventive health services, before the fear of COVID-19 sets in and disrupts and/or damages individuals’ psychological functioning. When developing resilience and hope intervention programs, if spatial distancing measures put in place to prevent the virus from spreading are taken into consideration, then programs suitable for online administration could be developed accordingly. Indeed, online positive psychology interventions have been shown to be effective in decreasing depression and anxiety and increasing good psychological well-being (Gander et al. 2016; Chakhssi et al. 2018). It may be beneficial to design intervention programs so that they may be adapted to be used in a face to face setting, once the risk of the spread of the virus has ceased. Moreover, resilience and hope program interventions in school settings have also demonstrated positive results regarding the development of physical and mental health (Edwards and McClintock 2013; Stallard and Buck 2013). The widespread use of such programs in all schools can be construed as a form of investment in strengthening individuals during the current COVID-19 climate and protection of mental health in the case of any future outbreaks that may occur. Mental health specialists such as psychologists, counselors, social workers, and psychiatric nurses may benefit from the findings of the research here with regard to the struggle in preventing the fear of COVID-19 and the protection of the mental health more generally in society.

### Limitations

There are some limitations present in the present study. Firstly, the data for the study were derived from self-report scales which suffer from well-known method biases. In collecting the data, the use of multiple methods would be beneficial in reducing bias in the answering of questions. Secondly, as the study design was cross-sectional, it is not possible to make definitive conclusions regarding the cause and effect correlations between the variables under investigation. Longitudinal studies are needed to determine the direction of the relationships. Thirdly, while the data were collected from participants across Turkey, the sample was not necessarily nationally representative, so the findings here would need replicating using larger more nationally representative studies both in and outside of Turkey. Finally, only two factors (i.e., hope and resilience) were examined as protective factors against the fear of COVID-19 and only subjective happiness, which is an indicator of mental health, was investigated in the study. For this reason, future research should examine other psychological strengths and attributes such as optimism, humor, creativity, spirituality, and vitality as protective factors, which may also have great utility in developing methods of protection against fear of COVID-19 and improving mental health.

## Conclusion

The protection of mental health due to the consequences of COVID-19 is threatening global health and is currently one of the matters of utmost psychological concern. In light of this, the findings of the present study clearly demonstrate that resilience and hope as protective factors are associated with decreased fear of COVID-19 and increased subjective happiness of individuals. In other words, it appears that individuals who have the strength to cope with stressful life situations and have hope that alternative solutions may be found are more able to cope with challenging life events (in this case, COVID-19). Their experience of fear can be dealt with adaptively at a practical and functioning level and consequently they are able to feel a greater sense of subjective happiness.
